# Influence of state of health and personality factors of resilience and coping in healthy subjects and those with diabetes

**DOI:** 10.3389/fpubh.2023.1074613

**Published:** 2023-03-02

**Authors:** Cristina Rivera-Picón, María Hinojal Benavente-Cuesta, María Paz Quevedo-Aguado, Raúl Juárez-Vela, Jesús Martinez-Tofe, Juan Luis Sánchez-González, Pedro Manuel Rodríguez-Muñoz

**Affiliations:** ^1^Faculty of Health Sciences, Nursing, Pontifical University of Salamanca, Salamanca, Spain; ^2^Nursing Department, Faculty of Health Sciences, Research Group GRUPAC, University of La Rioja, Logroño, Spain; ^3^Faculty of Nursing and Physiotherapy, University of Salamanca, Salamanca, Spain; ^4^Department of Nursing, Instituto Maimónides de Investigación Biomédica de Córboda, Córdona, Spain

**Keywords:** resilience, coping strategies, personality factors, diabetes mellitus, chronic diseases

## Abstract

**Introduction:**

Currently, the most common chronic metabolic disease in our society is Diabetes Mellitus. The diagnosis of Diabetes Mellitus supposes an impact for the patient, since it requires a modification in the lifestyle, which demands a great capacity for adaptation and modification of habits. The aim of the study was to determine whether personality factors and health status influence resilience and coping strategies in a sample of healthy and diabetic subjects.

**Methodology:**

The sample included a total of 401 subjects (201 patients with Diabetes and 200 without pathology). The instruments applied for data collection were: Sociodemographic data questionnaire, the Resilience Scale, the Coping Strategies Questionnaire and The “Big Five” factor taxonomy. The data collection period was approximately 2 years (between February 2018 and January 2020).

**Results:**

Certain personality factors, such as Emotional Stability, Integrity, Conscientiousness and Extraversion, were positively related to Resilience. Additionally, Emotional Stability, Integrity, and Extraversion were positively associated with Rational Coping. On the other hand, emotional stability, agreeableness and extraversion were negatively related to emotional coping. In relation to health status, the absence of pathology is related to the use of rational strategies more than to the diagnosis of diabetes. Therefore, the participants in this study present different psychological patterns depending on personality and health status.

**Conclusions:**

The present study shows that the subjects of the sample present different psychological patterns depending on Personality and health status.

## 1. Introduction

According to the latest studies, the most frequent chronic metabolic disease in our society is diabetes mellitus (1). In 2002, the WHO announced a worldwide prevalence of diabetes of 3%, which corresponds to 170 million people in the world diagnosed with this pathology. It was even estimated that this figure would double by 2025 (2). Today, these forecasts have already been exceeded. The latest figures provided by the International Diabetes Federation (IDF), corresponding to 2019, showed that 9.3% of adults have diabetes, which corresponds to a total of 463 million people. They also indicated that 1.1 million children and adolescents under the age of 20 live with type 1 diabetes. In addition, the IDF estimates that in 2030, 578 million adults will be living with the disease. In 2045, it is estimated that the figure will rise to 700 million (3).

These data are of great importance because the diagnosis, prognosis and treatment of the disease have a great emotional impact on the patient. This is associated with the need to assume a pathology that will accompany the subject throughout their life and the subject's obligation to modify their life habits in order to obtain a better quality of life, thus reducing complications deriving from it (4).

There is even research that details the psychological repercussions that accompany diabetes (5, 6). Thus, it has been stated that said pathology can be associated with depression and anxiety. These two diseases can arise regardless of the type of diabetes, especially in the presence of clinical complications. Therefore, it is essential that health workers use programmes in their clinical practice that address the emotional demands detected. Even the American Diabetes Association has incorporated new medical care recommendations, with the intention of including the assessment of the psychological and social situation of subjects diagnosed with diabetes (7, 8).

Based on the aforementioned theoretical aspects, some research has been oriented toward the identification of those psychological mediators that could contribute to achieving a better quality of life in subjects with chronic disease. Thus, the impact of resilience, personality and coping strategies on the health of subjects with chronic pathologies has been studied.

Therefore, research studying resilience and coping strategies has increased (9–11). Resilience is defined from the health field as the ability of individuals to maintain health and quality of life in a dynamic and challenging environment. Therefore, it is considered relevant variable for in the area of health due to its capacity to buffer stress (12–16). In the study by Pasantes et al. interventions were carried out with diabetic patients aimed at promoting their level of resilience. These incidents had a positive result on hemoglobin A1C levels (17). The type of coping strategies that people use to adapt to their illness can anticipate the impact caused by said illness. Therefore, certain coping styles can mediate and buffer the effects of stress. It is stated that active coping strategies are positively related to health (18).

In addition, personality may also play a fundamental role in the way subjects deal with the disease, directly influencing their wellbeing. Thus, personality modulates the way in which people face and adapt to a chronic disease, favoring the development of resilience and the use of coping strategies (19–21). The psychological aspect of diabetes is considered an important part of the treatment and management of this condition in the modern world. Thus, the assessment of personality traits can play a substantial role in the proper treatment of diabetics. The study by Esmaeilinasab et al. was determined that extraversion in diabetic patients is associated with better disease control (21). In addition, this may be relevant if we take into account that some studies indicate that patients with diabetes have different personality traits than subjects without pathology (22). In the context of this study, personality is approached through the Big Five model in Spanish, which hierarchically orders five personality factors: emotional stability, agreeableness, integrity, conscientiousness and extraversion (23).

Based on the scientific evidence outlined above, the objective of this study was to determine whether health status and personality factors influence resilience and coping strategies in a sample of healthy and diabetic subjects. For this reason, we consider it essential to know which diseases predict a worse adaptation, as well as those personality characteristics that favor the development of resilience, in order to focus on effective and individualized health programme.

The novelty of this study is justified in the use of a clinical sample. This allowed us to assess the influence of the Personality, but also the health status of the subjects to explain the Resilience and Coping. In most studies, only the relationships between these psychological variables have been investigated, through samples with healthy population, such as university students (24, 25).

## 2. Methodology

### 2.1. Aim and design of the study

The aim of this study was to determine whether health status and personality factors influence resilience and coping strategies in a sample of healthy and diabetic subjects. The study had a non-experimental cross-sectional design with a correlational objective.

### 2.2. Participants

**Figure 1:**
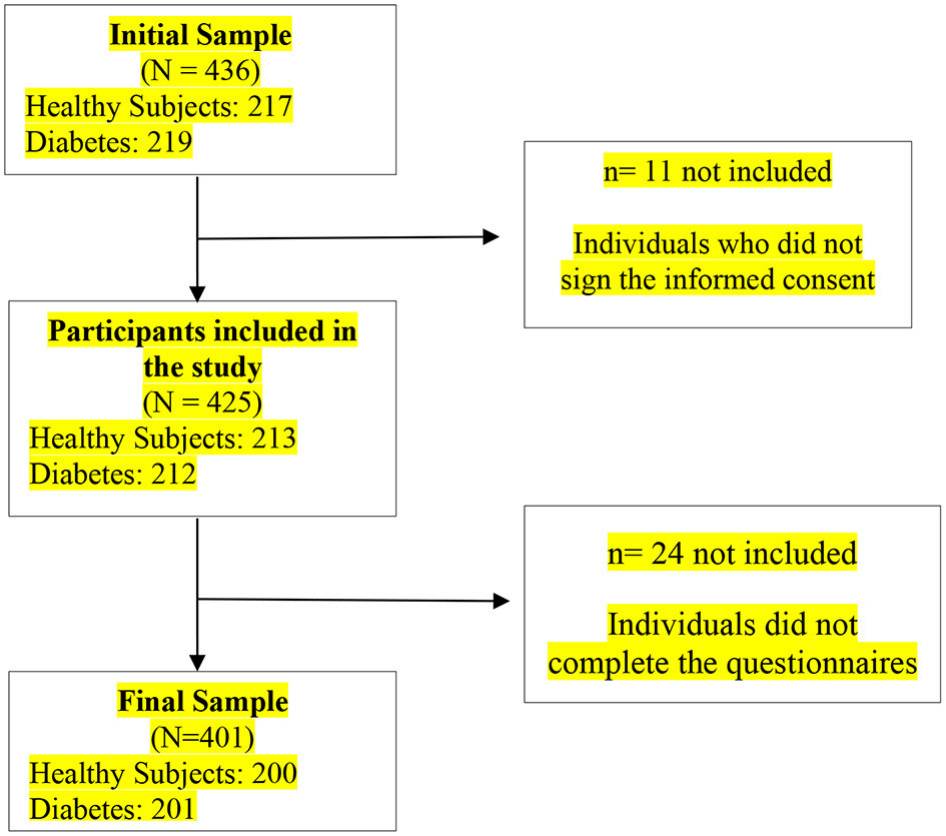
Sample selection flowchart.

These samples were selected at the University Assistance Complex of Salamanca. Four hundred and thirty six subjects participated in the study, of which 35 were excluded for not completing the informed consent or not completing the questionnaires. The total sample consisted of 401 subjects (Figure 1).

The study participants were 200 healthy subjects and 201 patients with diabetes (*N* = 401). The majority of it is made up of men (*N* = 285) and are mainly aged between 44 and 50 years (*N* = 118). Most of the subjects are married/in a couple (*N* = 247) and only 85 subjects have higher education (Table 1).

**Table 1:** Sample description.

	**Diabetes**		**Healthy**		**Total**		**Ji**	**TE**	***p***
	***N***	**%**	***N***	**%**	***N***	**%**			
N° participants	201	50.1%	200	49.9%	401	100%			
**Sex**									
Woman	58	28.9%	58	29.0%	116	28.9%	0.001	0.002	0.975
Man	143	71.1%	142	71.0%	285	71.6%			
**Age**									
43 years or younger	46	22.9%	59	29.5%	105	26.2%	2.588	0.080	0.460
44 to 50 years	63	31.3%	55	27.5%	118	29.4%			
From 51 to 55 years old	44	21,9%	38	19.0%	82	20.4%			
56 years or older	48	23,9%	48	24.0%	96	23.9%			
**Marital status**									
Married/couple	123	61.2%	124	62.0%	247	61.6%	4.574	0.107	0.102
Single/widowed/other	64	31.8%	51	25.5%	115	28.7%			
Separated/divorced	14	07.0%	25	12.5%	39	9.7%			
**Level of studies**									
Secondary or lower	160	79.6%	156	78.0%	316	78.8%	0.154	0.020	0.695
Superior	41	20.4%	44	22.0%	85	21.2%			

Pearson's χ2 test was used, using Cramer's V to determine the effect size. Thus, it was detected that the subsamples of our study, no significant differences were detected in the sociodemographic variables (*p* > 0.05) (Table 1).

In both subsamples, to participate in the project, the following inclusion criteria must be met: The subjects can be of legal age and participate voluntarily in the study. In the case of subjects with diabetes, an additional inclusion criterion was having a confirmed diagnosis of said disease, regardless of its stage. An additional inclusion criterion in healthy patients was that they were not diagnosed with any disease. The exclusion criteria were: suffering from a disease that would prevent the patient from completing the study, not agree to participate in the study and have been diagnosed with an affective pathology that could bias the results.

### 2.3. Data collection

The samples were selected following a quota sample with equivalent age ranges, sex and educational level, with the aim of achieving homogeneous sub-samples. The sample was collected at the University Assistance Complex of Salamanca. The selection of the subsample made up of diabetic patients was carried out in the Diabetes Unit of the Clinical Hospital of Salamanca and the Internal Medicine hospitalization wards of the same hospital.

After obtaining the sample of subjects with diabetes, the sample of healthy subjects was selected. The selection of this subsample was carried out in different Salamanca health centers (namely, “Periurbana Sur” and “Capuchinos” Health Centers). These patients voluntarily participated in the study after attending their scheduled appointment in the nursing consultation.

The data collection period was ~2 years (between February 2018 and January 2020), through the instruments detailed below.

#### 2.3.1. Sociodemographic data questionnaire

Sociodemographic data were collected through an instrument made up of a series of questions of a socio-demographic nature and information on the presence of diabetes.

- Health status (subjects without pathology, subjects with diabetes).
- Socio-demographic variables studied (age, sex, marital status and educational level).

#### 2.3.2. Personality: “Big Five” factor taxonomy

To assess personality factors, the “Taxonomic Proposal of the Big Five in Spanish” was produced by Iraegui and Quevedo-Aguado (26). This research consisted of a psycholinguistic approach to the study of personality following the “Big Five hypothesis.” Principal component factor analysis was applied to the 150 mini-markers finally identified in this research as personality descriptors. The Kaiser rule was employed to select the number of factors to retain and varimax normalization was used as the rotation method.

The five factor solution required ten iterations for convergence and explained 19.36% of the total variance with a Cronbach's α of 0.88. For our study we have used a reduced scale of 50 personality descriptors, ten for each factor (five positive and five negative). These descriptors were chosen based on their correlations with the corresponding factor. In this investigation, the use of the reduced version was chosen due to its brevity and its adequate psychometric properties. The global reliability of the instrument is α = 0.884, finding each of the five factors in indices that oscillate between α = 0.079 and α = 0.89 (26).

In this scale, the subjects have to evaluate these 50 descriptors depending on whether they are suitable or not for defining their personality traits. The response range was from 0, not suitable, to 4, very suitable. A total score was obtained for each of the factors.

#### 2.3.3. Coping strategies questionnarie

The Coping Strategies Questionnaire scale was designed by the authors Sandín and Chorot in 2002. This questionnaire contains a scale made up of 42 items, which score from 0 (never) to 4 (almost always). Through this scale, two general dimensions of coping can be measured: emotional coping and rational coping. Also, based on this general classification, it allows assessing seven more specific coping dimensions. Thus, emotional coping includes negative self-focused coping and overt emotional expression. Rational coping included problem-solving coping, positive reappraisal, and seeking social support. Each coping factor/dimension includes seven items, with the total variance explained by the seven factors being 55.3% (27).

#### 2.3.4. Resilience scale

Wagnild and Young created The Resilience Scale in 1993, adapted by Novella in 2002 into Spanish (28). This scale has 25 items. Each item ranges from 1 (strongly disagree) to 7 (strongly agree).

The scale assesses five resilience factors: personal satisfaction, equanimity, feeling good alone, self-confidence, and perseverance. Global internal consistency was measured using Cronbach's α coefficient (α = 0.88).

### 2.4. Ethical considerations

This study received a positive report from the Clinical Research Ethics Committee of the University Hospital of Salamanca PIO02/01/2018.

### 2.5. Data analysis

Statistical analysis was performed using International Business Machines' (IBM) Statistical Package for the Social Sciences (SPSS) version 25 (IBM Corp., Armonk, NY, USA). To determine whether personality factors and health status influence resilience and coping strategies in a sample of healthy subjects and those with diabetes, linear regression analysis was performed.

This technique requires the fulfillment of five assumptions: independence, non-collinearity, linearity, homoscedasticity and normality. After verifying these assumptions, the linear regression analysis was applied. For this, the variables were grouped into two blocks. One of them contained the dummy variables, and the other the rest of the variables. For the first block the method was introduced, while for the second the stepwise regression was obtained. To study the fit of the model, the coefficient of determination (*R*^2^) and the adjusted coefficient of determination ($R_{A}^{2}$) were used. In all statistical test, testing was significant when *p* > 0.05.

## 3. Results

### 3.1. Linear regression

For each dependent variable, a linear regression analysis was performed. These de-pendent variables (DVs) were resilience, rational coping and emotional coping. In contrast, the independent variables (IVs) were the factors of personality and the state of health of the subjects. In categorical VI with two or more levels, it was necessary to create dummy variables.

#### 3.1.1. Resilience

In relation to resiliene, the regression revealed that the best model is the one in which four variables were included (*R*^2^ = 0.848, $R_{A}^{2\;}=$ 0.719, F (df1, df2) = 20.047 (1, 393), *p* ≤ 0.001). These predictor variables included in the final model explained 71.6% of the variance in DVs.

Table 2 shows that emotional stability, integrity, conscientiousness and extraversion were positively related to resilience. Personality factor 1 (emotional stability) was the one with the greatest weight (B = 0.883, β = 0.457, t = 11.998, *p* < 0.001). In relation to health status, the dummy of healthy subjects was not significant (B = 0.990, β = 0.875, t = 1.137, *p* = 0.256).

**Table 2:** Final model coefficients: resilience.

	**B**	**Std error**	**β**	**t**	***p***
Constant	32.589	3.938		8.279	< 0.001
F1 emotional stability factor	0.883	0.074	0.457	11.998	< 0.001
F3 integrity factor	0.663	0.115	0.221	5.756	< 0.001
F4 conscientiousness factor	0.449	0.099	0.175	5.531	< 0.001
F5 extraversion factor	0.384	0.078	0.166	4.936	< 0.001
Healthy subjects	0.990	0.875	0.031	1.137	0.256

B, unstandardized regression coefficients; Std error, standard error; β, standardized regression coefficients; p, *p*-value.

Finally, the existence of atypical and predominant cases was assessed. Table 3 shows the value of the most extreme cases in different measures. Two atypical cases are detected in RV but neither of them is predominant.

**Table 3:** Atypical and predominant cases: resilience (linear regression).

	**Maximum**	**Minimum**	**Cases out of range**
Typified residues	4.166	−2.971	2
Waste diversion	4.288	−3.103	2
Leverage	0.075	-	0
Cook's distance	0.110	-	0

#### 3.1.2. Rational coping

The regression revealed that the best model is the one in which four variables were included (*R*^2^ = 0.776, $R_{A}^{2}$ = 0.598, F (df1, df2) = 22.527 (1, 393), *p* < 0.001). These predictor variables included in the final model explained 59.8% of the RV variance.

Table 4 shows that emotional stability, integrity and extraversion are positively related to rational coping. In relation to the state of health, the dummy of healthy subjects was significant, being the variable that had the greatest weight (B = 4.244, β = 0.153, *t* = 4.746, *p* ≤ 0.001).

**Table 4:** Final model coefficients: rational coping.

	**B**	**Std error**	**β**	**t**	***p***
Constant	23.016	2.702		8.521	< 0.001
F1 emotional stability factor	0.554	0.073	0.333	7.582	< 0.001
F3 integrity factor	0.859	0.101	0.331	8.507	< 0.001
F5 extraversion factor	0.491	0.080	0.247	6.161	< 0.001
Healthy subjects	4.244	0.894	0.153	4.746	< 0.001

B, Unstandardized Regression Coefficients; Std. error, standard error; β, Standardized Regression Coefficients; p, *p*-value.

Finally, the existence of atypical and predominant cases was assessed. Table 5 shows the value of the most extreme cases in different measures. Two atypical cases are detected, but neither is predominant.

**Table 5:** Atypical and predominant cases: rational coping (linear regression).

	**Maximum**	**Minimum**	**Cases out of range**
Typified residues	4.116	−2.971	2
Waste diversion	4.288	−3.103	4
Leverage	0.075	-	0
Cook's distance	0.110	-	0

#### 3.1.3. Emotional coping

The regression revealed that the best model is the one in which three variables were included (*R*^2^ = 0.457, $R_{A}^{2}$ = 0.243, F (df1, df2) = 5.539 (1, 394), *p* = 0.019). These predictor variables included in the final model explained 24.3% of the variance in DVs.

Table 6 shows that emotional stability, agreeableness and extraversion were negatively related to emotional coping. In relation to health status, healthy subjects have negative coefficients, but this variable was not significant (B = −0.375, β = 0.033, *t* = −0.733, *p* = 0.464).

**Table 6:** Final model coefficients: emotional coping.

	**B**	**Std error**	**β**	**t**	***p***
Constant	25.231	1.309		19.278	< 0.001
F1 Emotional Stability factor	−0.257	0.042	−0.378	−6.125	< 0.001
F2 Agreeableness factor	−0.181	0.048	−0.204	−3.783	< 0.001
F5 Extraversion factor	−0.106	0.046	−0.130	−2.323	0.021
Healthy subjects	−0.375	0.511	−0.033	−0.733	0.464

B, Unstandardized Regression Coefficients; Std. error, standard error; β, Standardized Regression Coefficients; p, *p*-value.

Finally, the existence of atypical and predominant cases was assessed. Table 7 shows the value of the most extreme cases in different measures. Two atypical cases are detected, but neither of them is predominant.

**Table 7:** Atypical and predominant cases: emotional coping (linear regression).

	**Maximum**	**Minimum**	**Cases out of range**
Typified residues	4.166	−2.973	2
Waste diversion	4.288	−3.103	3
Leverage	0.070	-	0
Cook's distance	0.110	-	0

## 4. Discussion

Different studies have confirmed that the diagnosis of a chronic pathology and individual differences in personality traits can influence the development and maintenance of resilience and coping strategies (20, 26–29). Then, these results are compared with those of our project.

The results obtained in our research show that emotional stability, integrity, responsibility and extraversion were positively related to resilience. We can highlight the fact that emotional stability turned out to be a significant predictor in all models. In addition, it was the variable with the highest weight for the resilience variable.

In a similar vein, numerous studies reflect the negative association between resilience and neuroticism (19, 25, 30–35). In order to compare this argument with the results of our re-search, it should be noted that the subjects with a low score in neuroticism are located on the opposite side of the emotional stability factor. Therefore, the results of the cited authors coincide with those of our study: the emotional stability factor was the one that was most related to higher levels of resilience.

Other research also reflects the positive relationship between extraversion, integrity and conscientiousness with levels of resilience (33, 35–39). This evidence also coincides with the results presented in our study, which reflect that the three factors contributed significantly to the prediction of resilience.

Also, the results of our research, in relation to the study of coping strategies, revealed that emotional stability, integrity and extraversion were positively related to rational coping. Emotional stability, agreeableness and extraversion were negatively associated with emotional coping. Other authors have also addressed the relationship between personality factors and coping strategies. The research found on this study topic indicates that the way in which an individual faces problem is influenced by their personality traits (40, 41). Thus, Mirnics et al. (42) found in their research that emotional stability was the trait that most significantly predicted coping strategies. Thus, emotional stability was associated positively with rational strategies and negatively with emotional strategies. In addition, extraversion and conscientiousness were found to be positively related to the use of rational strategies. Other authors, such as Afshar et al. (43), found similar results in their research, pointing out that subjects with a higher level of extraversion, integrity and emotional stability frequently use more rational strategies. This evidence is in line with the results obtained in our study. We also found results similar to those obtained in our research in the work of Leszko et al. (44), which indicated that agreeableness is negatively associated with emotional coping.

Therefore, the results obtained in our research are consistent with published studies on personality factors that predict resilience and coping. In relation to health status, our research shows that the absence of a pathology predicted a greater use of rational coping strategies.

However, there is little research focused on predicting the levels of resilience and coping strategies used based on the health status of the subjects. However, it has been described that the diagnosis of a chronic disease is a stress factor, hindering the development of resilience and threatening the coping capacity of the individual (14). Our results predicted that healthy and diabetic subjects would not present differences in the resilience variable. Thus, subjects with diabetes have learned how to face, overcome and transform themselves in the face of adversity.

It should be noted that there is very little research with which we can compare these results. Thus, few studies compare the level of resilience of subjects with diabetes and healthy subjects. However, we found similar results to those presented in our project in the research carried out by Novaes (45), which was conducted with a sample of subjects with diabetes mellitus and healthy subjects. In this study, it was found that there were no significant differences in the level of resilience between the groups. This finding coincides with that obtained in our study (45).

However, other studies have shown that healthy subjects have a higher level of resilience than patients with chronic pathologies (14). Thus, we consider it necessary to develop more research that evaluates and com-pares resilience in specific chronic diseases. It should be noted that each chronic disease has very different characteristics in terms of its development and therapeutic plan. For this reason, it is necessary to carry out more studies that compare the level of resilience according to the state of health. We consider it essential to investigate and learn about the pathologies associated with lower levels of resilience, since different studies coincide in believing that resilient people are more capable of coping with disease processes, both their own and those of others, and emerge stronger from the situation (13, 46).

Finally, the results of this study show that the state of health was also related to the type of coping strategies. The subsample of healthy subjects presented a greater use of rational cutting strategies. These strategies are associated with positive coping, coping with stress and trauma differently between individuals (47). Furthermore, rational coping, characterized by the mobilization of the patient to deal with the disease, is associated with greater adaptation to the disease and a higher quality of life (46–49). However, we find opposite conclusions in other studies, which state that diabetic subjects more frequently use rational coping strategies (50, 51).

### 4.1. Limitations

We point out as the main limitation that personality and health status only explained 24.3% of emotional coping. Therefore, variables that help improve our predictions are missing. However, there are investigations that state that resilience and gender can also predict the type of emotional coping used (52). Future research should take into account these variables not included in the models, which may be relevant for predicting the variables that are not well-explained.

## 5. Conclusion

Subjects present different psychological patterns depending on personality and health status. This conclusion may be useful in clinical practice for developing strategies, individually, focused on individuals with certain personality characteristics that predict a greater risk of maladjustment to their disease. Also, in an individualized way, strategies could be developed focused on individuals with certain personality characteristics that predict a greater risk of maladjustment to their disease. Therefore, the conclusions of this study show the importance of developing individualized health programs to address diabetes. However, it would be important to expand the study with other chronic diseases.

## Data availability statement

The original contributions presented in the study are included in the article/supplementary material, further inquiries can be directed to the corresponding author.

## Ethics statement

The studies involving human participants were reviewed and approved by El Comité Ético de Investigación Con Medicamentos del Área de Salud de Salamanca. Código CEIC: PlO02/01/2018. The patients/participants provided their written informed consent to participate in this study.

## Author contributions

Conceptualization: CR-P, MHB-C, and PMR-M; methodology: CR-P and JM-T; software: JM-T and JLS-G; validation: MHB-C, RJ-V, and CR-P; formal analysis: CR-P, RJ-V, and JLS-G; investigation: RJ-V and PMR-M; resources: PMR-M; data curation: CR-P, JLS-G, and JM-T; writing-original draft preparation: CR-P and PMR-M; writing-review and editing: CR-P and MPQ-A; visualization: CR-P and JM-T; supervision: JM-T and PMR-M; project administration: CR-P; funding acquisition: RJ-V, JM-T, and JLS-G. All authors have read and agreed to the published version of the manuscript.
